# Cooperation Between Photosynthetic and Antioxidant Systems: An Important Factor in the Adaptation of *Ulva prolifera* to Abiotic Factors on the Sea Surface

**DOI:** 10.3389/fpls.2019.00648

**Published:** 2019-05-21

**Authors:** Xinyu Zhao, Yi Zhong, Huanxin Zhang, Tongfei Qu, Yongshun Jiang, Xuexi Tang, Ying Wang

**Affiliations:** ^1^College of Marine Life Sciences, Ocean University of China, Qingdao, China; ^2^Laboratory for Marine Ecology and Environmental Science, Qingdao National Laboratory for Marine Science and Technology, Qingdao, China

**Keywords:** *Ulva prolifera*, thallus mat, photosynthetic system, antioxidant system, cooperation, spatiotemporal attribute

## Abstract

Large-scale green tides have occurred continuously in the Yellow Sea of China from 2007 to 2018, and the causative species of the Yellow Sea green tide (YSGT) is *Ulva prolifera*. The thalli form floated thallus mats, and the thalli from different layers of the thallus mat suffer significantly different environmental conditions. In the present study, the environmental conditions of the surface layer (SL), middle layer (ML), and lower layer (LL) of the thallus mat from mid-June (Stage I) to mid-July (Stage II) were simulated. Photosynthetic traits and antioxidant systems were measured. The results showed that (1) photoprotective [non-photochemical quenching (NPQ) and cyclic electron transport (CEF)] and antioxidant systems both play important roles in protecting against abiotic factors in *U. prolifera*. (2) Cooperation between NPQ and CEF was observed in the ML group; CEF and the antioxidant system in the SL group work synergistically to protect the thalli. Furthermore, an inferred spatiotemporal attribute regarding the YSGT is presented: the significant changes in abiotic factors on the sea surface can easily affect the thalli of SL and ML from mid-June to mid-July, and those of LL can be affected in mid-July. This cooperation combined with the spatiotemporal attributes offers an explanation for the annual occurrence of the YSGT.

HIGHLIGHTS

–Adaptive mechanisms of *Ulva prolifera* against abiotic factors.

–Cooperation between photosynthetic and antioxidant systems.

–Spatiotemporal attributes regarding the Yellow Sea green tide are presented.

## Introduction

The excessive growth of green algae, such as *Ulva, Chaetomorpha*, and *Cladophora*, has been reported as the reason for the formation of green tides globally and constitutes a growing environmental problem in eutrophicated coastal ecosystems (Merceron et al., 2007). Large-scale green tide in the Yellow Sea occurred continuously from 2007 to 2018 over a 10-year period. Ye et al. (2008) confirmed that the causative species of the YSGT is *Ulva prolifera*. The YSGT has various serious implications, such as the blocking of navigation channels in the immediate area of the bloom and local deposition on the shore, which can be destructive to estuary communities at different trophic levels or may cause economic losses to fisheries and tourism. YSGT thus constitutes a serious marine ecological disaster. The costs for clean-up and emergency responses to the 2008 bloom were estimated at between RMB 592 million (US$96 million) and RMB 2 billion (US$325 million), and consequential losses to the aquaculture production of sea cucumbers, cockles, and scallops were RMB 800 million (US$130 million) (Keesing et al., 2016).

The source area of the YSGT is in the southern Yellow Sea, where approximately 6500 t of *U. prolifera* thalli were released into the sea water. Liu et al. (2010) divided the period of YSGT into two stages (early stage and outbreak stage), and two cruises were conducted to analyze the morphological and genetic diversity of drifting *Ulva* thalli sampled from latitude 32–36° as well as culture-derived samples taken from surface water during the second cruise. Kim et al. (2011) divided the green tide thalli into two classes (late-stage vegetation and early-stage vegetation) in a laboratory study. When YSGT occurred, the thalli drifted northward from the source area and formed a massive floating green tide south of the Shandong Peninsula (from mid-April to late-May) (Huo et al., 2014; Wang et al., 2015). In a previous study, the occurrence period of the YSGT was divided into three stages (pre-bloom, bloom, and post-bloom) in a laboratory experiment (Wang et al., 2012). The long distance and long duration of the drift between the source area and the location of the bloom are important characteristics of the YSGT, and the time at which YSGT outbreak occurs is specific. The outbreak period of the YSGT is in June each year, and the post-bloom stage of the YSGT is accompanied by changes in the environmental conditions in July (Liu et al., 2013; Wang et al., 2015).

When the YSGT occurred, the thalli of *U. prolifera* floated on the sea surface and formed a thallus mat (Wang et al., 2015). Ivan et al. (1997) researched the ecophysiological and ecological consequences of the thallus mat, which was studied as a whole body. As the thallus mat can reach up to 0.5-m in thickness, the thalli at different layers from the top to the bottom of the mat experience significantly different environmental conditions, e.g., temperature, light intensity, and emersion/immersion (Lin et al., 2011). Changes in the abiotic factors affect the growth and metabolism of macroalgae (Mou et al., 2013). Lin et al. (2011) compared the photosynthetic parameters of different-colored floating thalli, and Zhao et al. (2016) studied the different photosynthetic response characteristics of the SL and LL in the floating thalli.

Photosynthesis is an important process that provides energy and a material basis for macroalgae. Abiotic factors of light, temperature, and emersion/immersion significantly impact the normal physiological activity of plants (Bernacchi and Long, 2002; Li et al., 2009; Gao et al., 2011). Excess light exceeds the photosynthetic adaptability of the thalli, and the absorption of excess light can lead to the increased production of highly reactive intermediates, potentially causing photo-oxidative damage and the inhibition of photosynthesis (Li et al., 2009). Macroalgae can resist stresses from the environment due to several stress-resistant physiological mechanisms, such as CEF and NPQ (Munekage et al., 2004; Li et al., 2009). CEF around PSI is more tolerant under desiccation stress than the electron flow around PSII (Lu et al., 2016), and PSII is sensitive, while PSI is relatively stable, under high irradiances and different saline stresses (Aro et al., 1993). Gao and Wang (2012) concluded that CEF in *Porphyra yezoensis* played an important role in desiccation and rehydration processes. PSI-driven CEF can provide desiccation tolerance for *Ulva* spp. (Gao et al., 2011). NPQ is another crucial mechanism that exists in macroalgae, and it works by dissipating excess energy in the LHCs as heat (Li et al., 2009). Zhang et al. (2013) concluded that both PSBS and LHCSR proteins were expressed in green algae, and these two proteins can induce NPQ protection mechanisms (Peers et al., 2009; Zhang et al., 2013).

Antioxidant metabolism also plays an important role in the ability of *U. prolifera* to adapt to different environmental obstacles, such as variability in salinity, solar radiation, and tidal variations (Smit, 2004; Connan et al., 2007). SOD, CAT, GPX, APX, and GR are members of the antioxidant system, and they play important roles in the adaptation of the thalli to the environment (Imlay, 2003). The influence of day-night and tidal cycles on the antioxidant capacities in three intertidal brown seaweeds, namely *Pelvetia canaliculata, Ascophyllum nodosum*, and *Bifurcaria bifurcata*, were studied, and the results showed that these three types of seaweeds all possessed relatively high levels of antioxidants (Connan et al., 2007). Luo and Liu (2011) concluded that antioxidants and ROS play an important role in the tolerance of salinity stress by *U. prolifera*. Dummermuth et al. (2003) concluded that macroalgae possess a high antioxidant capacity for coping with complex intertidal environmental changes.

A previous study, which was conducted *in situ*, showed that the thalli of *U. prolifera* can adapt to the environment on the sea surface through NPQ and CEF, while some other mechanisms that can work in conjunction with photosynthetic adaptation mechanisms might exist to protect the thalli (Zhao et al., 2016). The antioxidant system is also important for the clearing of ROS in *U. prolifera* (Luo and Liu, 2011). Similar working mechanisms between CEF and the antioxidant system may indicate a correlation between photosynthetic and antioxidant systems. In the present study, the environmental conditions of the SL, ML, and LL in the thallus mat were simulated during the bloom stage in the laboratory, and the response of photosynthetic and antioxidant systems in *U. prolifera* against the complex environment on the sea surface was analyzed. Additionally, an explanation from the perspective of the cooperation between photosynthetic and antioxidant systems in *U. prolifera* was studied to deepen the understanding of the adaptive mechanisms in the thalli of *U. prolifera*. Both the cooperation and spatiotemporal attributes of YSGT can explain the annual occurrence of the green tide from May to July in the Yellow Sea of China.

## Materials and Methods

### Species and Treatments

The experiment was undertaken on free-floating thalli of *U. prolifera* that were collected from coastal Qingdao in June 2016 during the bloom period. The thalli were cleaned gently with a brush, and sterile seawater was used to remove the attached sediment, small grazers, and epiphytes. The thalli were then cultured in sterile seawater, which was collected from the same location as the thalli of *U. prolifera* and enriched with f/2 medium. The culture temperature was set at 20°C, and light intensity was set at 72 μmol m^-2^ s^-1^ with 12:12 h light:dark cycle in a GXZ-280C intelligent illumination incubator. To inhibit the growth of diatoms, germanium dioxide (GeO_2_) at a concentration of 0.5 mg/L was used. Media were renewed every 2 days.

As the bloom and post-bloom periods of YSGT are in June and July each year, it is essential to study the responses of *U. prolifera* to the changes in abiotic factors from mid-June to mid-July in the Yellow Sea. The climatic conditions during the experiment are those typical from mid-June to mid-July in the region. The SST in the Yellow Sea changes significantly from late June (21°C) to early July (24°C) (Liu et al., 2009; Wang et al., 2015). The average daily air temperature is 23°C in late June and 27°C in early July (source: Weather China^1^). Light intensity in the Yellow Sea during the bloom is approximately 100 μmol m^-2^ s^-1^ in late June and 240 μmol m^-2^ s^-1^ in early July, which were used as the light level of SL (Gao et al., 2011, 2016; Keesing et al., 2016). The light level of LL was 5 μmol m^-2^ s^-1^ based on Zhao et al. (2016). The thalli of ML were designated as the transition between those of SL and LL, and the environmental conditions of the ML were the same as SL except for the volume of seawater added into the flasks (400 mL).

Based on the above information, the experiment was divided into two stages:

(1) Stage I: In this stage, the research simulated the process of outbreak in late June, and the thalli were placed in flasks [500 mL; one thallus per flask (4 g)] and cultured in GXZ-300C intelligent illumination incubators (Jiangnan, Ningbo, China) under two treatment conditions. The environmental conditions of the SL, ML, and LL thalli in the thallus mat in Stage I were marked as Stage I-SL, Stage I-ML, and Stage I-LL, respectively. The parameters of SST and air temperature were used to simulate the living temperature of the LL and SL thalli. For the SL group, 30 mL of seawater was added into each flask to simulate the thalli on the SL of the thallus mat, which could ensure that part of the thallus was exposed to air directly. The flasks required shaking every 4 h to simulate the effect of sea waves on the thalli of SL under natural conditions.
(2) Stage II: The process of outbreak in early July was simulated in this stage, and the experimental system was similar to that in Stage I. The abiotic factors of the SL, ML, and LL thalli in the thallus mat in Stage II were separately marked as Stage II-SL, Stage II-ML, and Stage II-LL.

**FIGURE 1:**
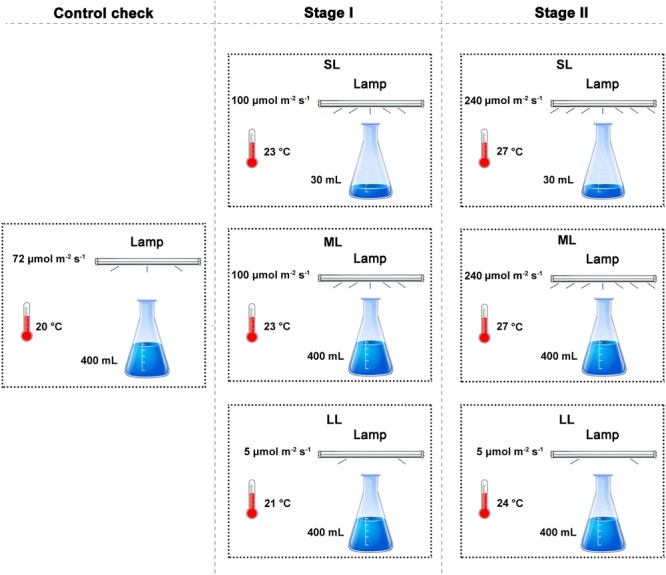
A sketch map of our study to simulate the living environment of the free-floating *Ulva prolifera*.

The experimental setup of Stage I and Stage II is shown in Table 1, and the test procedures are shown in Figure 1. The experimental period was 1 month (14 days for Stage I and 14 days for Stage II), and this period is the same duration as from late June to early July. Thalli samples were collected at 10:00 am at 24 h, 7, 14, 21, and 28 days to assess photosynthetic parameters and antioxidant systems. One-half of the thalli were used to conduct physiological measurements, while the other half was used to investigate the molecular-level responses of the floating thalli. To study the responses of *U. prolifera* to abiotic factors, a CK group was designed so that the results of the thalli of Stage I-SL, Stage I-LL, Stage II-SL, and Stage II-LL could be compared to the CK groups. The parameter design of the CK group is shown in Table 1, and the environmental conditions used constituted optimal conditions for the thalli (Wang et al., 2015; Gao et al., 2016; Keesing et al., 2016).

**Table 1:** The experimental setup of the simulated experiment in the laboratory.

	Temperature	PAR	Volume of
Group	(°C)	(μmol m^-2^ s^-1^)	seawater (mL)
CK	20	72	400
Stage I-SL	23	100	30
Stage I-ML	23	100	400
Stage I-LL	21	5	400
Stage II-SL	27	240	30
Stage II-ML	27	240	400
Stage II-LL	24	5	400

### Measurement of Chlorophyll Fluorescence

Photosynthetic performance was measured using a Dual-PAM-100 fluorometer (Walz, Germany) after the thalli were recovered. Before the experiments, the thalli were placed in the dark for 15 min. The settings of the Dual-PAM-100 fluorometer were based on a previous study (Zhao et al., 2016). Automated induction and recovery curve routine were used, and repetitive application of saturation pulses was used to obtain the related parameters of Chl fluorescence. The F_0_ and the *F*_m_ were determined after a saturating pulse of white light using the low irradiance of the red measuring light (approx. 0.15 μmol photons m^-2^ s^-1^). The parameter *F*_v_/*F*_m_, which can represent the PSII maximum quantum yield in the thalli, can be calculated by the equation: *F*_v_/*F*_m_ = (*F*_m_-*F*_0_)/*F*_m_, and this parameter can be used to evaluate the photosynthetic activity of *U. prolifera*. The [Y(II)] is also an important indicator for evaluating photosynthetic activity in the thalli. Y(NPQ) represents the Y(NPQ), and Y(NO) is the parameter that reflects the Y(NO) (Kramer et al., 2004). Relatively higher results of Y(NPQ) and Y(NO) can indicate that the thalli suffer greater negative effects from environmental stress, and higher values of Y(NPQ) indicate that *U. prolifera* possesses the physiological regulatory mechanisms to protect itself. To study the energy distribution between PSI and PSII, the response change in ratio of Y(I) to Y(II) can be used to estimate the activation of PSI-driven CEF (Harbinson and Foyer, 1991; Huang et al., 2013).

The method to determine the RLCs was based on a previous study (Zhao et al., 2016). RLCs can reflect the photosynthetic ability of the thalli, and the rETR of PSII can be determined by determining the RLCs. The RLCs were then fitted using the Platt’s empirical equation, and the parameter α (photosynthetic rate in light-limited region of the RLC) could be obtained (Platt et al., 1980).

### Inhibitor Treatment

The inhibitor DCMU can block electron transport after the primary acceptor plastoquinone QA (Joët et al., 2002), and the inhibitor was used to further study the PSI-driven CEF in the thalli. When the thalli were treated with the inhibitor (10 mM DCMU), the thalli were incubated at room temperature for 5 min. A Dual-PAM (Walz) was used to determine the CEF-related parameters.

### Analysis of Lipid Peroxidation and H_2_O_2_ Content

Thalli (0.1 g FW) were ground to a powder in liquid nitrogen, following which 1 mL 5% (w/v) trichloroacetic acid (TCA) was added to the ground thalli. To determine the contents of lipid peroxidation and H_2_O_2_, the mixture was centrifuged at 12,000 *g* for 10 min at 4°C.

The contents of H_2_O_2_ were determined using H_2_O_2_ kits according to the manufacturer’s instructions (Nanjing Jiancheng, China). The level of lipid peroxidation was determined using TBA-reacting substance contents, and the content of MDA was measured (Buege and Aust, 1978). TBA [0.2 mL, 0.6% (v/v)] was added to each 0.2 mL aliquot of supernatant, following which the mixture was heated at 95°C for 40 min. The mixture was then rapidly cooled in an ice bath. The absorbance of the supernatant was finally recorded at 532 nm and 450 nm after centrifugation at 4,000 *g* for 10 min.

### Determination of Antioxidant Content

To measure the content of ascorbate, the samples (0.1 g FW) were ground using liquid nitrogen, and 1 mL of 2.5 M HClO_4_ was mixed with the thalli. The extracts were neutralized to pH 5.6 with 1.25 M K_2_CO_3_ and centrifuged at 5,000 *g* for 10 min after incubation on ice for 30 min. Fifty microliters of thallus sample, 5 U mL^-1^ ascorbate oxidase, 700 μL sodium phosphate buffer (pH = 5.6), and 1% (w/v) PVP-40 were combined, and the ascorbate content was determined spectrophotometrically by recording the absorbance at 265 nm (Foyer et al., 1983).

Samples (0.1 g FW) were homogenized in liquid nitrogen, and then 5% (v/v) 5-sulphosalicylic acid containing 1% (w/v) PVP-40 was used to extract total glutathione (reduced and oxidized). Twenty-five microliters of sample, 700 μL reaction buffer, 100 μL 6 mM 5,5′-dithio-bis (2-nitrobenzoic acid), 175 μL distilled water, and 25 μL 266 U mL^-1^ GR were mixed, and the content of glutathione was determined by recording the absorbance at 412 nm at 30°C. The results were quantified using a standard curve (Anderson, 1985).

### Analysis of Antioxidant Enzyme Activity

Thalli (0.1 g FW) were ground in liquid nitrogen, and then the samples were extracted with 1 mL 0.05 M potassium phosphate buffer (pH = 7.0), which contained 0.25% (v/v) Triton X-100 and 1% (w/v) PVP-40, followed by centrifugation for 10 min at 12,000 *g* at 4°C. The supernatant was used to measure total soluble protein (TSP) and the activity of antioxidant enzymes (SOD, GPX, APX, and CAT).

The content of TSP was determined using the Coomassie blue dye binding assay (Bradford, 1976), and the standard curve of protein quantities could be determined by the protein solution kits according to the manufacturer’s instructions (Nanjing Jiancheng, China).

SOD activity was measured using the method of Cyt *c* reduction by Mishra et al. (1993). One unit of SOD activity was defined as the amount of SOD that inhibited the rate of Cyt *c* reduction by 50% under experimental conditions. GPX activity was measured according to Lawrence and Burk (1976) and was calculated from the initial reaction rate after deducting the non-enzyme oxidation. One unit of GPX activity was defined as the amount of enzyme required to reduce 1 μmol GSH per minute. APX activity was measured according to (Nakano and Asada, 1981). The reaction mixture contained the supernatant, 0.05 M potassium phosphate buffer (pH = 7.0), 0.15 mM ascorbate, and 0.3% H_2_O_2_ (hydrogen peroxide). Absorbance at 290 nm after 1 min of the reaction was measured. One unit of APX activity is defined as the amount necessary to decompose 1 μmol ascorbate per min.

Catalase activity was measured according to Dhindsa et al. (1981). Potassium phosphate buffer (1.5 mL at 50 mM, pH = 7.0), 1 mL deionized water, 0.3 mL 0.1 M H_2_O_2_, and the extract were mixed at 25°C. CAT activity was measured by recording the absorbance at 240 nm using a spectrophotometer. One unit of CAT activity was defined as the amount of enzyme required to decrease 0.1 absorbance unit in the optical density at 240 nm per min.

### Analysis of Gene Expression by Quantitative Real-Time PCR

Quantitative real-time PCR (qRT-PCR) was performed using an ABI StepOnePlus^TM^ real-Time PCR System (ABI, United States) and SGExcel FastSYBR Mixture (Sangon Biotech, China) according to the manufacturer’s instructions. Tublin (*Tub*) was chosen as the reference gene, and a 112-bp product of *Tub* was amplified with the primers *Tub*-F (5′-CAAGGATGTCAATGCTGCTGT-3′) and *Tub*-R (5′-G ACCGTAGGTGGCTGGTAGTT-3′) (Table 2; Wu et al., 2009). To detect the transcript abundance of genes related to NPQ and antioxidant defense enzymes, the full-length cDNAs of the *Lhc*SR (GenBank no. HQ889161), *Psb*S (GenBank no. JX625151), *Sod* (GenBank no. EF437244), and *Cat* (GenBank no. ABB88582) genes were used to design the forward and reverse primers for the qRT-PCR: 5′-GCATTTGTG AGGCATACCG-3′ and 5′-TTACCAGTTCTTGTGCGACG-3′for *Lhc*SR, 5′-AAC AGGTTCATCCATCACGG-3′ and 5′-TTGCCTCAAACTCATCCTCTG-3′ for *Psb*S, 5′-TATATCTCCGCTGAGATCATGG-3′ and 5′-TTGTTGTAGTTGGTGACATACG-3′ for *Sod*, and 5′-GAATACCTTGACCAAAGTGGTT-3′ and 5′-GTAAGTGCAGTC TACGTCG-3′ for *Cat* (Table 2; Wu et al., 2009; Dong et al., 2012). For these experiments, qRT-PCR was performed in triplicate for each sample, and dissociation curve analysis of the amplification products was performed at the end of each PCR reaction to confirm that only one specific PCR product had been amplified and detected. The 2^-ΔΔCT^ method was used to analyze the relative expression levels of the genes (Livak and Schmittgen, 2001).

**Table 2:** Quantitative real-time PCR primers used in this study.

			Melting
			temperature	Size of
Gene	Nucleotide sequence (5′→3′)		of product	product
*Tub*	Forward	CAAGGATGTCAATGCTGCTGT	58.4	112
	Reverse	GACCGTAGGTGGCTGGTAGTT		
*Lhc*SR	Forward	GCATTTGTGAGGCATACCG	57.0	221
	Reverse	TTACCAGTTCTTGTGCGACG		
*Psb*S	Forward	AACAGGTTCATCCATCACGG	58.0	104
	Reverse	TTGCCTCAAACTCATCCTCTG		
*Sod*	Forward	TATATCTCCGCTGAGATCATGG	60.0	71
	Reverse	TTGTTGTAGTTGGTGACATACG		
*Cat*	Forward	GAATACCTTGACCAAAGTGGTT	76.7	95
	Reverse	GTAAGTGCAGTCTACGTCG		

### Statistical Analysis

All experiments were performed in triplicate. The three replicates were used for the measurement of chlorophyll fluorescence and inhibitor treatment, and the results of lipid peroxidation, H_2_O_2_ content, antioxidant content, antioxidant enzyme activity, and qRT-PCR data were obtained by analyzing the three biological replicates of the thalli. The results were tested in a one-way ANOVA using SPSS 22.0 statistical software (IBM Corp., Armonk, NY, United States), and Bivariate Pearson’s correlation analysis was performed to assess the relationships of the parameters for photosynthetic and antioxidant systems in *U. prolifera*. The figures related to this study were generated using SigmaPlot^TM^ 12.5 software (Systat Software Inc., United States).

Principal components analysis (PCA) was used as an exploratory tool to obtain an overall measure of the thalli of *U. prolifera* on the basis of the simultaneous analysis of 18 responses to abiotic factors on the sea surface. Correlations are removed by PCA and attributes are converted into new, combined variables. This multivariate approach is considered to be particularly suitable for datasets with possible correlations among attributes (Manly and Alberto, 2017). The resulting independent principal components (PCs) are ranked by PCA according to their importance in explaining the variation in the dataset, and the resulting correlation coefficients facilitate the distinction of key traits characterizing the environmental response in each dimension. The thalli samples that cluster close to either end of a particular axis can then be related to responses that are positively or negatively correlated with a particular PC axis. Another aim for using PCA was to identity constitutive thalli attributes that are correlated with the principle environmental response (PC1). The PCA analysis was conducted using SPSS 22.0.

## Results

### Analysis of *F*_v_/*F*_m_, Y(II), and Y(NPQ) in *U. prolifera*

**FIGURE 2:**
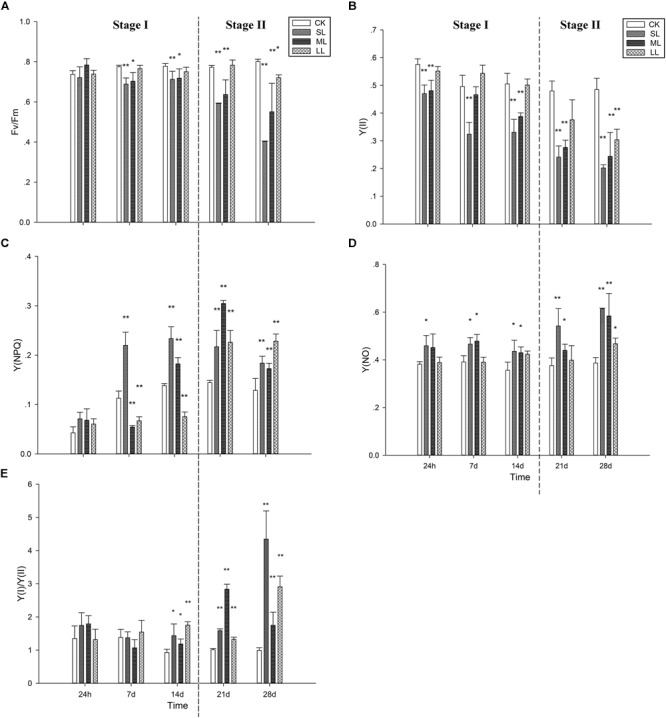
Variations of **(A)** *F*_v_/*F*_m_, **(B)** the effective quantum yield of PSII [Y(II)], **(C)** the quantum yield of the regulated energy dissipation in PSII [Y(NPQ)], **(D)** the quantum yield of non-regulated energy dissipation in PSII [Y(NO)], and **(E)** the ratio of Y(I) to Y(II) in *U. prolifera* under different climate conditions. ^∗^Indicates significant difference between treatment and CK groups (*P* < 0.05). ^∗∗^Indicates significant difference between treatment and CK groups (*P* < 0.01). Experiments were performed in triplicate, and the data are the mean of three independent experiments (±SD).

The changes in *F*_v_/*F*_m_ during the course of the experimental period are presented in Figure 2A. No significant changes were observed between the CK, SL, ML, and LL groups on the first day (one-way ANOVA, *P* > 0.05). The *F*_v_/*F*_m_ of the SL (one-way ANOVA, *P* < 0.01) and ML group (one-way ANOVA; *P* < 0.05) decreased significantly in contrast to the CK group, and no significant changes were observed between the CK group and the LL group on day 7 (one-way ANOVA; *P* > 0.05). The trend of the results on day 7 and day 14 was similar, and more significant changes between the ML group and the CK group were observed on day 21 (Figure 2A) (one-way ANOVA, *P* < 0.01). The values of *F*_v_/*F*_m_ for the SL group and ML group still remained at a relatively low level, and the value of *F*_v_/*F*_m_ in the thalli of the LL group decreased significantly in contrast to the CK group on day 28 (one-way ANOVA, *P* < 0.05). For Y(II), similar trends as *F*_v_/*F*_m_ were observed.

The changes in Y(NPQ) are shown in Figure 2C. No significant difference was observed between the four groups (one-way ANOVA, *P* > 0.05). An obvious increasing tendency in the SL group was observed after day 7 (one-way ANOVA, *P* < 0.01). Although the Y(NPQ) of the ML group decreased significantly on day 7 (one-way ANOVA, *P* < 0.01), a clear increase was detected after day 14, and the peak value appeared on day 21 (one-way ANOVA, *P* < 0.01). The Y(NPQ) of the LL group on day 7 and day 14 was significantly lower than that of the CK group (one-way ANOVA, *P* > 0.05), which increased markedly after day 21. Regarding Y(NO), the SL group increased significantly before day 14 (one-way ANOVA, *P* < 0.05) (Figure 2D). No significant differences in Y(NO) were observed between the CK group and the ML group at 24 h, but significant increases were detected on days 7, 14, and 21 (one-way ANOVA, *P* < 0.05) as well as on day 28 (one-way ANOVA, *P* < 0.01; Figure 2D).

Figure 2E shows that no significant changes in the ratios of Y(I)/Y(II) were observed between the CK, SL, ML, and LL groups at 24 h and 7 days (one-way ANOVA, *P* > 0.05). The Y(I)/Y(II) ratios of the SL, ML, and LL groups all increased significantly on day 21 and day 28 (one-way ANOVA, *P* < 0.01). The increase in Y(I)/Y(II) in the ML group was obviously higher than that of the SL group on day 21, and the opposite trend was found on day 28.

### Analysis of Rapid Light Curves (RLCs) of *U. prolifera*

**FIGURE 3:**
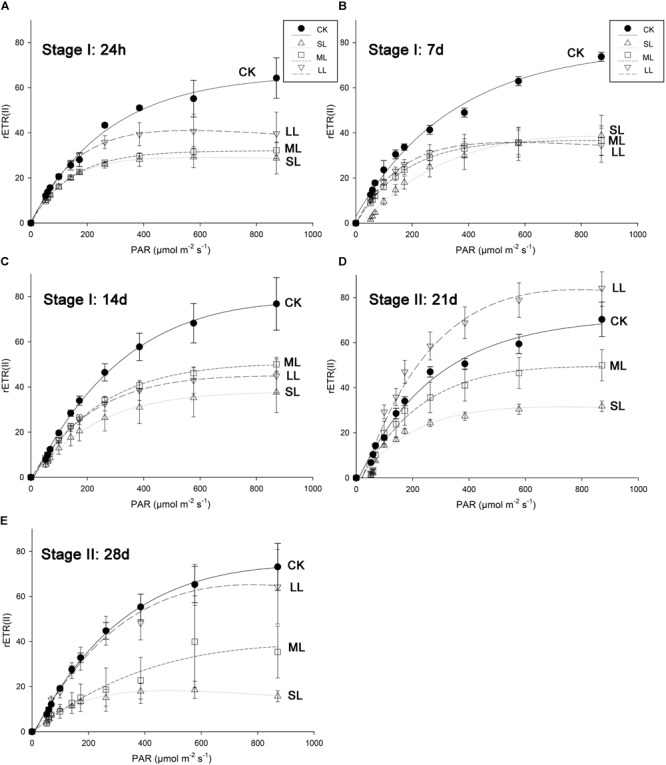
Mean relative electron transport rate (rETR) of rapid light response curves (RLCs) in *U. prolifera* of CK, SL, ML, and LL at **(A)** 24 h, **(B)** 7 days, **(C)** 14 days, **(D)** 21 days, and **(E)** 28 days. All experiments were performed in triplicate. The data are the mean of three independent experiments (±SD).

**FIGURE 4:**
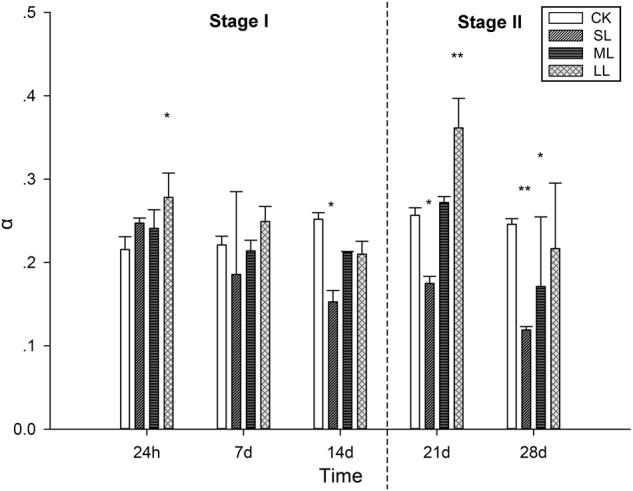
Variations in the parameters of the RLCs in *U. prolifera* under different climate conditions. ^∗^Indicates significant difference between treatment and CK groups (*P* < 0.05). ^∗∗^Indicates significant difference between treatment and CK groups (*P* < 0.01). All experiments were performed in triplicate. The data are the mean of three independent experiments (±SD).

Further conclusions can be drawn by analyzing the results of the RLCs, and the mean rETR of the RLCs in the thalli of *U. prolifera* at 24 h, 7, 14, 21, and 28 days are presented in Figure 3. No significant changes in the CK, ML, and LL groups were observed during the experiment, and a significant inhibitory effect in the SL group of the rETR(II) was observed as the experiment progressed (Figure 3). The parameter α can be used to detect light use efficiency (Platt et al., 1980), and the results of α can be seen in Figure 4 of this report. Significant differences between the CK group and the LL group were detected at 24 h (one-way ANOVA, *P* < 0.05), and α increased significantly at 21 days (one-way ANOVA, *P* < 0.01). The parameter α decreased significantly at 14 days, and a greater downtrend could be seen at 28 days (one-way ANOVA, *P* < 0.01).

### Responses to Inhibitors of ETR(I)

Table 3 reveals that ETR(I) in the thalli of the CK group did not change significantly throughout the experiment (one-way ANOVA, *P* > 0.05), and no significant difference was observed among the CK, SL, ML, and LL groups during the first 24 h (one-way ANOVA, *P* > 0.05). No significant difference was detected between the CK, ML, and LL groups, whereas ETR(I) in the thalli of the SL group increased significantly compared to the CK group on day 7 (one-way ANOVA, *P* < 0.01). ETR(I) of the thalli of the SL group still maintained a relatively high level, and the value of ETR(I) of the ML group increases significantly on day 14 (one-way ANOVA, *P* < 0.01). A slightly decreasing trend in ETR(I) of the SL group was observed (one-way ANOVA, *P* < 0.01), and ETR(I) in the ML group increased substantially (one-way ANOVA, *P* < 0.01).

**Table 3:** ETR(I) of the thalli and of the thalli treated with the inhibitor DCMU.

			ETR (I) (DCMU)/
Group	ETR(I) (no inhibitor)	ETR(I) (DCMU)	ETR (I) (no inhibitor)
24 h-CK	16.93 ± 4.31	3.56 ± 0.69	22.23%
24 h-SL	17.93 ± 3.32	4.16 ± 0.25	23.94%
24 h-ML	18.77 ± 3.39	4.09 ± 0.19	22.37%
24 h-LL	15.87 ± 3.93	3.92 ± 0.10	25.53%
7 days-CK	17.90 ± 1.81	5.15 ± 0.98	28.81%
7 days-SL	24.07 ± 0.25^∗∗^	6.58 ± 0.58	27.34%
7 days-ML	15.96 ± 3.55	4.85 ± 0.92	31.29%
7 days-LL	18.30 ± 4.67	4.52 ± 0.47	25.99%
14 days-CK	18.50 ± 3.08	5.20 ± 0.92	28.08%
14 days-SL	29.90 ± 3.80^∗∗^	10.36 ± 0.64^∗∗^	35.02%
14 days-ML	28.90 ± 3.17^∗∗^	9.06 ± 0.60^∗∗^	31.46%
14 days-LL	19.20 ± 1.51	7.84 ± 1.04	41.02%^∗^
21 days-CK	19.53 ± 4.20	6.01 ± 1.21	31.50%
21 days-SL	20.73 ± 2.66	10.17 ± 1.87^∗∗^	50.24%^∗∗^
21 days-ML	70.37 ± 4.01^∗∗^	32.40 ± 2.30^∗∗^	46.21%^∗∗^
21 days-LL	26.27 ± 2.11	10.77 ± 0.96^∗∗^	41.13%^∗∗^
28 days-CK	25.97 ± 1.59	7.26 ± 1.82	27.76%
28 days-SL	78.90 ± 12.44^∗∗^	45.57 ± 1.86^∗∗^	58.57%^∗∗^
28 days-ML	23.10 ± 0.92	11.35 ± 2.01^∗∗^	49.07%^∗∗^
28 days-LL	79.40 ± 4.64^∗∗^	35.29 ± 2.10^∗∗^	44.58%^∗∗^

*The ratio of CEF to total electron flow was calculated as ETR(I) (treated by DCMU)/ETR(I) (no inhibitor). Three biological replicates were measured, and the data are mean values from three independent measurements (±SD). ^∗^Indicates significant difference between treatments and control groups (*P* < 0.05). ^∗∗^Indicates significant difference between *in situ* and control groups (*P* < 0.01, one-way ANOVA).*

DCMU was used to inhibit LEF, and the values of ETR(I) decreased when the thalli were treated with the inhibitor DCMU. It can be seen in Table 3 that ETR(I) (DCMU) of the CK, SL, ML, and LL groups did not change significantly throughout the first 7 days (one-way ANOVA, *P* > 0.05). ETR(I) (DCMU) of the SL and ML groups increased significantly compared to the CK group (one-way ANOVA, *P* < 0.01), while no significant difference between the CK and LL groups was observed on day 14 (one-way ANOVA, *P* > 0.05). An upward trend in ETR(I) (DCMU) of the SL and LL groups was observed on days 21 and 28 (one-way ANOVA, *P* < 0.01), whereas ETR(I) (DCMU) of the ML group reached a peak on day 21 and decreased significantly on day 28 (one-way ANOVA, *P* < 0.01).

No significant difference in the ratio of ETR(I) (DCMU)/ETR(I) (no inhibitor) in the SL, ML, and LL groups was observed compared to the CK group (one-way ANOVA, *P* > 0.05). The ratio of the LL group increased on day 14 (one-way ANOVA, *P* < 0.05) and increased rapidly on days 21 and 28 (one-way ANOVA, *P* < 0.01). The values of ETR(I) (DCMU)/ETR(I) (no inhibitor) of the SL and ML groups significantly increased on days 21 and 28 (one-way ANOVA, *P* < 0.01).

### Analysis of the Antioxidant System of *U. prolifera*

**FIGURE 5:**
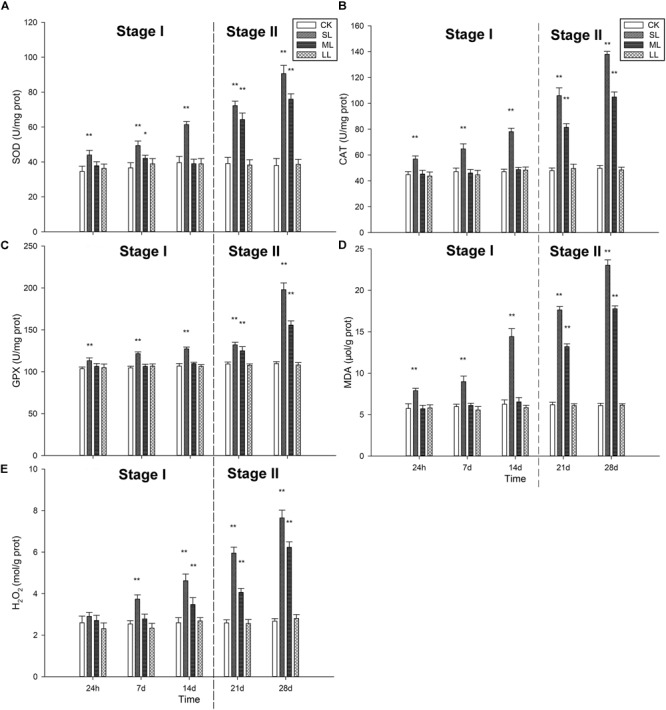
Changes in the antioxidant system in the thalli of *U. prolifera* under different climate conditions: **(A)** SOD activity, **(B)** CAT activity, **(C)** GPX activity, **(D)** MDA content, and **(E)** H_2_O_2_ content. ^∗^Indicates significant difference between treatment and CK groups (*P* < 0.05). ^∗∗^Indicates significant difference between treatment and CK groups (*P* < 0.01). All experiments were performed in triplicate. The data are the mean of three independent experiments (±SD).

The effects of different abiotic factors on the antioxidant system are shown in Figure 5. SOD activity was significantly (one-way ANOVA, *P* < 0.01) increased in the SL group compared to the CK group, and an increasing trend was seen throughout the experiment (Figure 5A). The activity of the ML group significantly increased on day 7 (one-way ANOVA, *P* < 0.05), but decreased on day 14. SOD activity demonstrated an increasing trend in the ML group on day 21 and day 28, and the values of the ML group were significantly lower than those of the SL group on day 21 and day 28 (Figure 5A).

Catalase activity exhibited a similar trend among the treatment groups compared to SOD activity (Figure 5B). The activity of the SL group was significantly higher than the other groups, and an increasing trend was observed during the experiment (Figure 5B). The CAT activity of the ML group did not change significantly during Stage I, but increased significantly on day 21 and day 28 (one-way ANOVA, *P* < 0.01). GPX activity in the SL group did not change significantly on days 1, 7, 14, and 21 (one-way ANOVA, *P* < 0.01), but increased on day 28. GPX activity in the SL group was significantly higher than the other groups throughout the experiment, except for day 21 (one-way ANOVA, *P* < 0.01). Changes in the ML group could be seen on days 21 and 28.

The content of MDA is shown in Figure 5D. Generally, the MDA content was significantly higher in the SL group than the other groups (one-way ANOVA, *P* < 0.01), and an increasing trend in the ML group was observed on days 21 and 28. H_2_O_2_ content exhibited different trends compared to MDA content. No significant difference in H_2_O_2_ content was observed in the four groups on the first day (one-way ANOVA, *P* > 0.05), and H_2_O_2_ content increased significantly after day 14 (one-way ANOVA, *P* < 0.01). A significant increasing trend in the SL group was detected after the first day, and H_2_O_2_ content in the SL group was higher than the other groups from day 7 to day 28 (Figure 5E).

### Expression Profiles of *Lhc*SR, *Psb*S, *Sod*, and *Cat*

**FIGURE 6:**
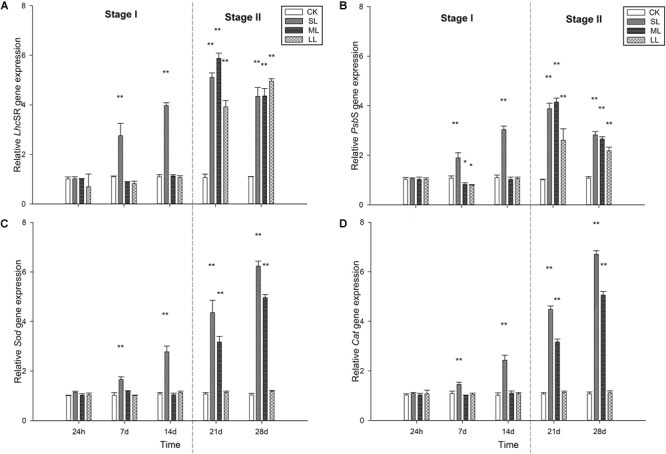
Changes in expression of the **(A)** *Lhc*SR, **(B)** *Psb*S, **(C)** *Sod*, and **(D)** *Cat* genes in the thalli of *U. prolifera* under different climate conditions. ^∗^Indicates significant difference between treatment and CK groups (*P* < 0.05). ^∗∗^Indicates significant difference between treatment and CK groups (*P* < 0.01). All experiments were performed in triplicate. The data are the mean of three independent experiments (±SD).

No significant difference in the transcript level of the *Lhc*SR gene was detected among the CK, ML, and LL groups on days 1, 7, and 14 (one-way ANOVA, *P* > 0.05), while that of the SL group increased significantly on days 7 and 14 (one-way ANOVA, *P* < 0.01). A clear increasing trend in the transcript levels of the *Lhc*SR gene in the SL, ML, and LL groups was observed during the experiment (one-way ANOVA, *P* < 0.01). Interestingly, the transcript levels in the ML group were significantly higher than those of the SL and the LL groups on day 21 (one-way ANOVA, *P* < 0.01). No significant difference between the SL and ML groups was seen on day 28, and that of the LL group increased to the highest level compared to the other two groups. A similar trend was observed in the transcript level of the *Psb*S gene compared to the *Lhc*SR gene on days 1, 7, 14, and 21, and the results of the SL and ML groups were both higher than that of the LL group (one-way ANOVA, *P* < 0.01, Figure 6).

Changes in the transcript levels of the *Sod* gene were similar to those of the *Cat* gene throughout the experiment. No significant difference was seen among the CK, SL, ML, and LL groups at 24 h (one-way ANOVA, *P* > 0.05), and the transcript levels of the two genes in the thalli of the LL group did not change significantly throughout the experiment (one-way ANOVA, *P* > 0.05). The transcript levels of both the *Sod* and *Cat* genes in the thalli of the SL group increased rapidly on days 7, 14, 21, and 28 (one-way ANOVA, *P* < 0.01), and the two genes in the thalli of the ML group started to increase significantly on day 21 (one-way ANOVA, *P* < 0.01, Figure 6).

### Plant Trait Responses in the First PC

**FIGURE 7:**
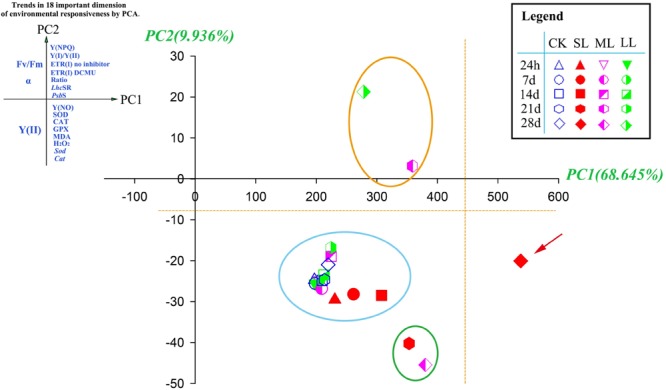
Principal components analysis (PCA) of 18 photosynthetic and antioxidant responses in CK, SL, ML, and LL thalli of *U. prolifera*. Plant responses associate with the first two dimensions (Table 4) are grouped at the end of each principal component, PC1 (*x*-axis) and PC2 (*y*-axis). Ratio: ETR(I) DCMU/ETR(I) no inhibitor.

Principal components analysis proved useful in exploring the changes that contributed to the main response patterns among the 20 thalli samples. High scores on PC1 were characterized by the antioxidant system, followed by CEF- and NPQ-related data. In addition, photosynthetic activity-related results tended to group on the negative PC1 axis (Table 4 and Figure 7).

**Table 4:** Correlation coefficients and probabilities for the thalli of *U. prolifera* attributes linked to the first two principal components (PCs).

	Correlation	Correlation
Trait description	with PC1	with PC2
*F*_v_/*F*_m_	–0.631	0.351
Y(II)	–0.919	–0.026
Y(NPQ)	0.643	0.454
Y(NO)	0.792	–0.371
Y(I)/Y(II)	0.754	0.004
ETR(I) no inhibitor	0.638	0.524
ETR(I) DCMU	0.752	0.409
ETR(I) DCMU/ETR(I) no inhibitor	0.782	0.180
α	–0.555	0.467
SOD	0.959	–0.123
CAT	0.965	–0.148
GPX	0.918	–0.230
MDA	0.960	–0.134
H_2_O_2_	0.945	–0.195
*Lhc*SR	0.801	0.494
*Psb*S	0.794	0.440
*Sod*	0.959	–0.144
*Cat*	0.955	–0.155

*Differences between means were regressed against the scores of PC1 and PC2.*

## Discussion

Previous research has evaluated the photosynthetic response of floating *U. prolifera* to *in situ* environmental changes on the sea surface, and typical abiotic factors (temperature, PAR, and emersion/immersion) during the outbreak of the YSGT have been identified (Zhao et al., 2016). This study provides the first multivariate assessment of the various intraspecific responses of *U. prolifera* thalli to changes in abiotic factors on the sea surface during the bloom and post-bloom of YSGT. Specifically, these results clearly showed the responses of the photosynthetic system and antioxidant metabolism of *U. prolifera*, and the relationships between photosynthetic and antioxidant systems could be evaluated in future research. The spatiotemporal attributes of the YSGT were studied, and the photosynthetic and antioxidant systems in the bloom and post-bloom thalli at different layers showed different response characteristics.

### Photosynthetic System

Environmental changes on the sea surface can affect the thalli, and the thalli possess several mechanisms to resist the environmental stress. Photosynthesis is an important process in *U. prolifera*, and several stress-responsive mechanisms make it possible for the thalli to resist stress, e.g., NPQ and CEF (Munekage et al., 2004; Li et al., 2009). The two stress-resident mechanisms NPQ and CEF have been widely found in various plants, such as vascular plants, mosses, and algae (Li et al., 2000; Munekage et al., 2004; Koziol et al., 2007; Gerotto et al., 2011). NPQ acts by dissipating excess energy in the LHCs as heat to ensure that the thalli are adapting to the environmental changes (Li et al., 2009). CEF around PSI is an important factor in the adaptation of macroalgae to dramatically changing environments on the sea surface, such as changes in temperature, light, saline stress, desiccation, and rehydration (Gao and Wang, 2012; Gao et al., 2013, 2015; Gylle et al., 2013).

In the present study, the photosynthetic activity of the SL and ML groups decreased slowly in the course of Stage I and decreased rapidly during Stage II, especially the SL group. Correspondingly, the Y(NPQ) in the SL and ML groups increased during the course of Stage I and decreased during Stage II. Y(NO) in the three groups was relatively stable during the bloom period and increased dramatically during the post-bloom period. This increase was particularly remarkable in the SL group. Based on the physiological experiments, molecular-level research was carried out using qRT-PCR, and the results were further analyzed by correlation analysis. Supplementary Table S2 shows that there was a high correlation (Supplementary Table S1; one-way ANOVA, *P* < 0.01) among the *Lhc*SR gene, *Psb*S gene, and Y(NPQ). This implies that the NPQ mechanism plays an important role in the floating thalli of *U. prolifera*.

In addition to the NPQ mechanism, the CEF mechanism also plays an important role in the floating thalli of *U. prolifera* (Zhao et al., 2016). The ratio of Y(I)/Y(II) of the SL, ML, and LL groups began to increase rapidly after 14 days from the start of the experiment, particularly in the SL group. Data on CEF in our present study can further confirm the present results.

### Antioxidant System

Previous studies have shown that the antioxidant system is also important for clearing the ROS in the thalli of *U. prolifera* (Imlay, 2003; Smit, 2004; Connan et al., 2007). SOD, CAT, and GPX are all important antioxidant system members, and relatively higher antioxidant capacity means higher adaptability (Dummermuth et al., 2003; Luo and Liu, 2011).

The results of the present study are consistent with previous conclusions, and the antioxidant system in the thalli of the SL group is especially important (Figure 5). In the SL group, the negative correlation between antioxidant enzymes and *F*_v_/*F*_m_ was higher than that of the CK group, especially for GPX activity (Supplementary Table S1, one-way ANOVA, *P* < 0.01). Furthermore, the negative correlation between antioxidant enzymes and Y(II) in the SL group was significantly higher than that of the CK group (Supplementary Table S1, one-way ANOVA, *P* < 0.01), which is indicative of low relative photosynthetic activity and positive antioxidant activity in the SL group. In the LL group, no significant correlation was detected between MDA, H_2_O_2_, SOD, CAT, and GPX, suggesting that the antioxidant system in the LL group is not effective. In the ML group, a significant positive correlation between MDA, H_2_O_2_, CAT, and GPX was observed, and as Figure 5D,E indicate, the content of MDA and H_2_O_2_ in the ML group was relatively higher than the LL group. The relatively higher content of MDA and H_2_O_2_ and the significant positive correlation between MDA, H_2_O_2_, CAT, and GPX indicate that CAT and GPX play an important role in clearing the oxidative stress in the thalli of the ML group. The positive correlation between MDA, H_2_O_2_, SOD, CAT, and GPX in the SL group was higher than the ML and LL groups, and this suggests that, in contrast to the ML group, SOD in the SL group works to protect the thalli (Supplementary Table S1, one-way ANOVA, *P* < 0.01). Hence, it is suggested that the antioxidant system plays an important role in the thalli of SL and ML, especially for the SL group. Furthermore, CAT and GPX play more important roles in protecting the thalli of *U. prolifera* under low environmental stress, and the three types of enzymes work together to resist extreme environments. This can further explain why the thalli possess such strong environmental adaptability during outbreaks of YSGT.

### Cooperation Between Photosynthetic and Antioxidant Systems

Many studies have confirmed that photosynthetic and antioxidant systems ensure the strong environmental adaptability of the thalli of *U. prolifera*. However, as a complete organism, the various components in the thalli work together, but little research has focused on the relationship between the photosynthetic and antioxidant systems. We studied the correlation between the two systems using correlation analysis and PCA (Supplementary Tables S1, S2).

The positive correlation between the activity of antioxidant enzymes and Y(I)/Y(II) was significantly higher than that of the CK group, and a similar result was observed between the activity of antioxidant enzymes and the ratios of ETR (I) (DCMU)/ETR (I) (no inhibitor) (Supplementary Table S1, one-way ANOVA, *P* < 0.01), which indicates a positive correlation between antioxidant system and CEF in the SL and CK groups. There was no significant correlation between antioxidant enzymes and CEF in the ML and LL groups (Supplementary Table S1, one-way ANOVA, *P* > 0.05). In the ML group, a significant increasing positive correlation was observed between Y(NPQ) and Y(I)/Y(II) (Supplementary Table S1, one-way ANOVA, *P* < 0.05), suggesting a positive correlation between NPQ and CEF in the ML group. Interestingly, there was no significant correlation between Y(NPQ) and CEF in the SL and LL groups (Supplementary Table S1, one-way ANOVA, *P* > 0.05), while a significant positive correlation existed between Y(NO) and Y(I)/Y(II) in the SL and LL groups (Supplementary Table S1, one-way ANOVA, *P* < 0.01). This indicates that both photochemical energy conversion and protective regulatory mechanisms in the SL and LL groups are inefficient, especially for the SL group (Kramer et al., 2004). These results further prove that the thalli in the ML group can adapt well to the changing environment on the surface because of NPQ and CEF. The environment of the SL thalli was worse than the ML and LL groups, and CEF and the antioxidant system in the thalli of the SL work synergistically to protect the thalli.

This study also provides a multivariate assessment of the various responses of *U. prolifera* to environmental changes on the sea surface. The most important dimension in describing the overall environmental response (PC1) contained all of photosynthetic and antioxidant responses of the thalli [Y(NPQ), Y(I)/Y(II), ETR (I) no inhibitor, ETR (I) DCMU, ETR(I) DCMU/ETR(I) no inhibitor, *Lhc*SR, *Psb*S, Y(NO), SOD, CAT, GPX, MDA, *Sod*, and *Cat*] (Figure 7), indicating the integrating nature of these traits as an overall summary for the individual effects caused by environmental changes on the sea surface. Decreases in photosynthetic activity were associated with increases in attributes linked to NPQ, CEF, and antioxidant activity (Table 4). Interestingly, all of the samples tended to group in the positive direction of the *x*-axis in the Figure 7, which is indicative of relatively low photosynthetic activity and active self-protection measures (NPQ, CEF, and antioxidant system), especially for the point of SL-28 days, and suggests relatively higher environmental stress upon the thalli (Lin et al., 2011; Zhao et al., 2016).

### Spatiotemporal Attributes of the YSGT

The YSGT outbreak occurs in June each year, and the YSGT enters the post-bloom stage in July and is accompanied by changes in environmental conditions (Liu et al., 2013; Wang et al., 2015). Light, temperature, and emersion/immersion all showed significant effects on algae and all constitute typical abiotic factors exhibited during the outbreak of the YSGT (Bernacchi and Long, 2002; Li et al., 2009; Gao et al., 2011; Zhao et al., 2016). Previous studies accurately recorded the changes in temperature and light intensity during the outbreak of the YSGT, and these data can be used as important references for this study (Liu et al., 2009; Gao et al., 2011, 2016; Wang et al., 2015; Keesing et al., 2016; Zhao et al., 2016). The response characteristics of *U. prolifera* to typical abiotic changes (light, temperature, and emersion/immersion) were studied in the laboratory by simulating the typical abiotic factors exhibited during the outbreak of the YSGT (Figure 1).

An inferred spatiotemporal attribute regarding the YSGT was presented. This means that the occurrence of the green tide has its own characteristics in time and space. In time, the green tide forms near the south of the Shandong peninsula in early June and can last until July. According to the changing characteristics of the environmental factors over time, this time period can be further divided into Stage I and Stage II. In space, the floating algae can form a thallus mat, which can be up to 0.5 m thick, during the outbreak of the green tide. The thalli in the SL, ML, and LL of the thallus mat were in different environments, and the photosynthetic system and antioxidant system of the thalli of the three locations showed different response characteristics. Spatiotemporal attributes can explain at physiological and molecular levels why the green tide recurs annually from May to July in the Yellow Sea of China. A massive free-floating green tide formed near the south of the Shandong peninsula in early June, and lasted until July (Liu et al., 2013; Wang et al., 2015). In the present study, the photosynthetic activity in the LL group was relatively stable during Stage I, and a significant downward trend could be observed until day 28 (one-way ANOVA, *P* < 0.05). However, a significant downward trend in the SL and ML groups was observed at Stage I in late June (one-way ANOVA, *P* < 0.01), and a rapidly declining trend was observed at Stage II in early July (one-way ANOVA, *P* < 0.01), which suggests that the thalli of the SL and ML in the thallus mat suffer more abiotic stresses (Figure 2). The bloom stage of the YSGT occurs from mid-June to mid-July, and significant changes in temperature and PAR exist during this period. The significant changes in temperature and PAR can easily affect the thalli of the SL and ML in the thallus mat, while those of the LL can be affected in mid-July.

## Conclusion

**FIGURE 8:**
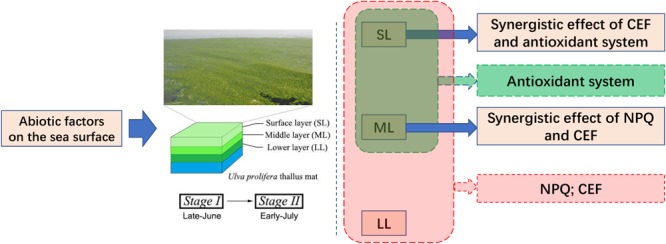
Schematic of the conclusion of our present study.

Significant changes in abiotic factors occur on the sea surface from Stage I (late-June) to Stage II (early-July), and *U. prolifera* is capable of adapting to the severe abiotic changes. Different abiotic adaptation strategies exist in the thalli of SL and ML, and synergistic effects occur in LL during the outbreak of the YSGT: (1) NPQ and CEF both play important roles in protecting the thalli. (2) The antioxidant system plays a significant role in protecting the thalli of SL and ML. (3) NPQ and CEF work synergistically to protect the thalli of the ML group, and the synergy between CEF and the antioxidant system has an important function in protecting *U. prolifera*. Furthermore, the spatiotemporal attributes of YSGT were inferred. Abiotic factors can easily affect the thalli of SL and ML from mid-June to mid-July, and those of LL can also be affected by abiotic factors in mid-July. The synergy between the photosynthetic and antioxidant systems of the YSGT, as well as the spatiotemporal attributes, can explain why the green tide recurs annually from May to July in the Yellow Sea of China (Figure 8).

## Author Contributions

XZ and YW conceived and designed the experiments. XZ and YZ performed the experiments, analyzed the data, and wrote the manuscript. XZ, YW, XT, HZ, TQ, and YJ contributed to reagents, materials, and analysis tools.

## Conflict of Interest Statement

The authors declare that the research was conducted in the absence of any commercial or financial relationships that could be construed as a potential conflict of interest.

## Footnotes

1 http://www.weather.com.cn/

## Supplementary Material

TABLE S1 Pearson’s correlation coefficients in the test index in the CK, SL, ML, and LL groups of *Ulva prolifera*.

TABLE S2 Pearson’s correlation coefficients in the test index in the thalli of *Ulva prolifera* on days 1, 7, 14, 21, and 28.

## Abbreviations

APX: ascorbate peroxidase
CAT: catalase
CEF: cyclic electron flow
CK: control check
Cyt *c*: cytochrome *c*
DCMU: 3-(3′,4′-dichlorophenyl)-1,1-dimethylurea
*F*_0_: initial fluorescence
*F*_m_: maximal fluorescence
*F*_v_/*F*_m_: maximum quantum yield of PSII
FW: fresh weight
GPX: glutathione peroxidase
GR: glutathione reductase
LHCs: light-harvesting complexes
LL: lower layer
MDA: malondialdehyde
ML: middle layer
NPQ: non-photochemical quenching
PAR: photosynthetically active radiation
PCA: principal components analysis
PSI: photosystem I
PSII: photosystem II
PVP-40: polyvinylpyrrolidone-40
qRT-PCR: quantitative real-time PCR
rETR: relative electron transport rate
RLCs: rapid light curves
ROS: reactive oxygen species
SL: surface layer
SOD: superoxide dismutase
SST: sea surface temperature
TBA: thiobarbituric acid
Y(NO): quantum yield of non-regulated energy dissipation in PSII
Y(NPQ): quantum yield of the regulated energy dissipation in PSII
Y(II): effective quantum yield of PSII
YSGT: Yellow Sea green tide
